# Hemorheological Alterations in Patients with Heart Failure with Reduced Ejection Fraction Treated by Resveratrol

**DOI:** 10.1155/2020/7262474

**Published:** 2020-06-30

**Authors:** Roland Gal, Dora Praksch, Peter Kenyeres, Miklos Rabai, Kalman Toth, Robert Halmosi, Tamas Habon

**Affiliations:** ^1^Division of Cardiology, 1st Department of Medicine, Medical School, University of Pécs, Hungary; ^2^Szentágothai Research Centre, University of Pécs, Hungary

## Abstract

**Objectives:**

Several beneficial effects of resveratrol have already been published. This study evaluated the effect of resveratrol on the hemorheological parameters in patients with heart failure with reduced ejection fraction.

**Methods:**

In our double-blind, placebo-controlled human clinical trial, we enrolled 60 outpatients with heart failure. Patients were randomized into two groups: receiving either 100 mg resveratrol capsule daily or placebo for 3 months. Hematocrit was determined by microhematocrit centrifuge. Plasma and whole blood viscosity was evaluated by capillary viscometer. Erythrocyte aggregation was measured by both LORCA and Myrenne aggregometers. LORCA ektacytometer was used for measuring erythrocyte deformability. Exercise capacity was assessed by a 6-minute walk test.

**Results:**

Resveratrol treatment did not have any significant effect on hematocrit and viscosity. The erythrocyte deformability also remained unchanged. However, significant improvement of red blood cell aggregation was observed in the resveratrol group compared to baseline after 3 months. Furthermore, positive correlation was found between the exercise capacity and the hemorheological properties (Hct, WBV, and RBC aggregation and deformability) as well.

**Conclusion:**

These findings indicate that resveratrol can significantly reduce red blood cell aggregation, which may positively influence microcirculation, which may contribute to the improvement of tissue perfusion and oxygen supply in heart failure.

## 1. Introduction

Heart failure (HF) continues to be a significant cause of cardiovascular mortality. Over the past few decades, numerous medical and device-based therapies have been developed for the management of heart failure; however, mortality remains high even in optimally treated patients [1]. Heart failure is a systemic, multifactorial disease, in which complex structural, neurohumoral, cellular, and molecular changes lead to volume overload, increased sympathetic activity, and redistribution of circulation and result in different, developing clinical signs and symptoms in parallel [2, 3]. Complex impairment of peripheral and coronary blood flow in HF including restricted microcirculation, attenuated regulatory mechanisms, and impaired hemorheological properties causes reduced oxygen utilization contributing to the symptoms and progression of heart failure [4–6].

Red blood cell (RBC) aggregation and deformability have an important role in capillary blood flow including coronary microcirculation. Besides many clinical states (e.g., ischemic heart disease, diabetes, and venous thrombosis), heart failure is known to be associated with increased RBC aggregation, which has a negative influence on the in vivo flow dynamics of blood. The reduction of RBC aggregation may have a positive effect on the flow properties of blood, which can be beneficial in cardiovascular diseases [7–10]. Furthermore, when the vascular autoregulatory reserve is exhausted in heart failure, the hemorheological disturbances—which can be easily compensated in healthy individuals—will have deleterious effects. Moreover, rheological disorders were found to be present even in the early stage of cardiovascular diseases, before their massive functional manifestation [5, 6, 11].

Over the past several decades, numerous reports have demonstrated enhanced expression of inflammatory cytokines (e.g., TNF-*α*, IL-1, IL-6, and IL-18) and reactive oxygen species (ROS) in chronic heart failure. These pathological conditions contribute to cardiac damage (remodelling, hypertrophy, fibrosis, etc.) and peripheral vascular disturbances [12–14]. Red blood cells are also prone to oxidative stress being the first cells in the body to be exposed to stressful stimuli, which results in abnormalities in the function, morphology, and metabolism of erythrocytes [15]. The aggregation and deformability of RBCs depend on the cellular properties as well as the mechanical and physicochemical characteristics of their environment (e.g., fibrinogen, large molecular weight globulins) [16], and both are influenced by the aforementioned processes.

French people tend to have a lower incidence of cardiovascular diseases despite having similar coronary risk factors as people in other industrialized countries. This phenomenon is known as the French paradox and is attributed to the higher red wine intake by the French [17].

Red wine contains high amount of polyphenolic compounds like resveratrol (RES), catechin, and quercetin, and RES is considered to be primarily responsible for the cardioprotective effect of red wine. Resveratrol (3,5,4-trihydroxystilbene) is a nonflavonoid polyphenolic compound produced by plants in response to environmental stress. Several mechanisms may be responsible for the cardioprotective effect of RES including reduction of oxidative stress, inflammation, and pathologic hypertrophic signaling and improved Ca^2+^ handling [18–22]. As these are important factors also in the pathogenesis of heart failure, we supposed that resveratrol may also have protective effects in heart failure.

The effects of moderate red wine consumption and the effectiveness of RES treatment on hemorheological parameters have already been evaluated in some previous animal and human trials. In a previous in vivo study of our working group, moderate red wine consumption had beneficial effect on red blood cell aggregation and deformability in healthy volunteers. Moreover, a significant improvement of RBC deformability and platelet aggregation was observed after RES administration (10 mg daily for 3 months) in patients with chronic ischemic heart disease [8, 23, 24].

According to literature, no effects of RES on rheological properties have been reported in heart failure. Therefore, the aim of this study was to test our hypothesis that resveratrol can improve the hemorheological parameters in patients with heart failure with reduced ejection fraction.

## 2. Materials and Methods

### 2.1. Study Design

**Figure 1:**
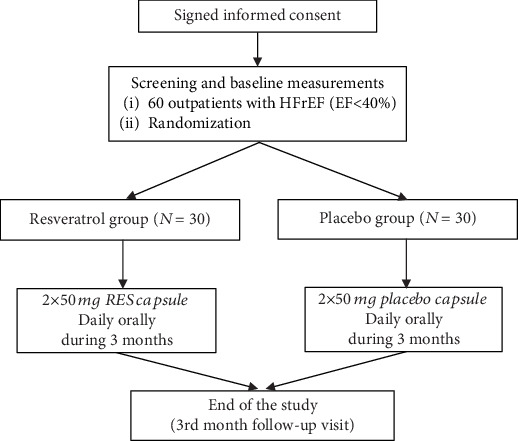
Study design.

Our trial was a single-center, double-blind, randomized placebo-controlled human study. The study design is summarized in Figure 1. The study was conducted in accordance with the principles stated in the Declaration of Helsinki (1996) and International Conference on Harmonization Good Clinical Practice, as well as local and national regulations. The protocol of the trial was approved by the Regional Ethics Committee of the University of Pécs (license number 5830). Written informed consent has been provided by all patients prior to any study-related procedures.

### 2.2. Eligibility

60 stable outpatients (age: 66.7 ± 2.01 years, 17 women and 43 men) with heart failure with reduced ejection fraction (HFrEF) in NYHA (New York Heart Association) class II or III were enrolled between 01/03/2016 and 30/11/2017 into our study (ejection fraction (EF) < 40%; ischemic/nonischemic: 34/26). They were randomized into two groups (resveratrol group and placebo group): 100 mg resveratrol capsules were administered orally for 3 months (2 × 50 mg) in the first group (*n* = 30) and identical placebo capsules in the second group (*n* = 30). The baseline values of RES and placebo groups were compared to the age-matched control group (mean age: 67.15 ± 1.01 years, female/male: 11/9), without heart failure (ejection fraction > 50%), and with moderate cardiovascular risk profile.

The resveratrol capsule and the matching placebo were purchased from ARGINA Nutraceuticals Ltd. (Fót, Hungary). The resveratrol capsule is commercially available and has official license for being marketed.

The main exclusion criteria were acute cardiovascular or cerebrovascular event, major cardiac surgery or intervention within 30 days prior to randomization, renal failure (estimated glomerular filtration rate (eGFR) < 20 ml/1.73 m^2^/min), or hepatic impairment (alanine aminotransferase (ALT) or aspartate aminotransferase (AST) ≥ 2x upper limit of normal (ULN) at baseline).

All of the involved patients received the evidence-based drug treatment for heart failure with reduced ejection fraction (HFrEF), including angiotensin-converting enzyme (ACE) inhibitors (or angiotensin receptor blocker (ARB)), beta-blockers, mineralocorticoid receptor antagonists (MRA), and in certain cases ivabradine. No patients were on angiotensin receptor-neprilysin inhibitor (ARNI) therapy. The preventive drug regime and the used doses were based on the actual ESC (European Society of Cardiology) heart failure guideline [2].

The patients had baseline and 3-month follow-up visits. During visits, the compliance of the patients was checked according to self-report and counting the remaining capsules at the final (3-month follow-up) visit. During the whole study period, subjects were in stable clinical condition and received unchanged medical therapy (Table 1).

The baseline characteristics and the homogeneity of our randomized population are described in Table 1. There were no significant differences in epidemiological characteristics between the placebo- and RES-treated groups at baseline.

### 2.3. Hemorheological Parameters

In this hemorheological substudy, blood samples were taken on the day of randomization and after 3 months from the antecubital vein after a 12-hour fasting. Blood samples for hemorheological measurements were collected into 2 × 6 ml ethylenediaminetetraacetic acid- (EDTA-) coated Vacutainer tubes with a 21-gauge butterfly infusion set. Hemorheological measurements were carried out within 2 hours after blood sampling [25].

The following hemorheological parameters were determined: hematocrit, plasma and whole blood viscosity, and red blood cell deformability and aggregation.

#### 2.3.1. Hematocrit

Hematocrit (Hct) was measured by Haemofuge microhematocrit centrifuge (Heraeus Instr.; Germany). Measurements were performed at room temperature (22 ± 1°C) [26].

#### 2.3.2. Plasma and Whole Blood Viscosity

Plasma viscosity (PV) and whole blood viscosity (WBV) values were determined by a Hevimet 40 capillary viscometer (Hemorex Ltd., Hungary). Plasma was collected after blood sample centrifugation for 10 minutes at 1500 g. Apparent WBV values interpolated to 90 s^−1^ shear rate were reported. Measurements were performed at 37°C [26].

#### 2.3.3. Red Blood Cell Aggregation

Red blood cell aggregation measurements were carried out with a LORCA aggregometer (Laser-assisted Optical Rotational Cell Analyzer; R&R Mechatronics, Hoorn, The Netherlands) based on syllectometry. 1 ml of oxygenated blood is injected into the gap between a static bob and a rotating cylinder that creates a simple shear flow. Erythrocytes are first disaggregated at high shear rate (500 s^−1^) then the shearing is stopped. The intensity of backscattered laser light is plotted against time on a syllectogram. The aggregation behavior of blood sample is characterized by the aggregation index (AI) calculated from the first 10 seconds of the syllectogram after the shape recovery period and by the time that elapses until intensity is reduced to half of the peak amplitude (*t*_1/2_). The smallest shear rate required for complete disaggregation (*γ*: gamma (1/s)) was also determined [27].

RBC aggregation was measured also by Myrenne aggregometer (MA-1 Aggregometer, Myrenne Ltd., Germany), applying the light transmission method of Schmid-Schönbein et al. This method calculates the aggregation index according to the change in intensity of transmitted infrared light during aggregation either at zero shear (*M*_0_) or low shear (*M*_1_ at 3 s^−1^) after disaggregation [28].

#### 2.3.4. Red Blood Cell Deformability

For the deformability measurement with LORCA ektacytometer, 25 *μ*l blood was suspended in high viscosity (32.6 mPas) polyvinylpyrrolidone solution. RBCs were sheared by shear stress from 0.3 Pa to 30 Pa, and their deformation was visualized by laser diffraction. The isointensive points of the diffraction pattern draw an ellipse with a longer *A* and a shorter *B* diameter. Deformation is characterized by the elongation index calculated by (*A*–*B*)/(*A* + *B*). Measurements were performed at 37°C. The deformability results were also analyzed by means of the Lineweaver-Burke nonlinear equation, with calculation of the maximal elongation index (EI_max_) at theoretical infinite shear and the shear stress value required for half of this maximal elongation (SS_1/2_) [29]. However, since goodness-of-fit results proved to be below the acceptable level in several cases and rejecting such cases would have biased results, we only used the raw elongation index (EI) parameters rather than EI_max_ and SS_1/2_ in the final analysis.

### 2.4. Six-Minute Walk Test

The six-minute walk test (6MWT) is a submaximal exercise test that measures the walking distance for 6 minutes. The test was performed on a 30 m long section of the corridor in our department according to the guideline of American Thoracic Society. The patients were in rest comfortably for 10 minutes prior to the test. The supervisor was a study nurse, who was blinded to the protocol. After 6 minutes or if the patient could not walk any further, the test was stopped and the distance and the reason for stopping (dyspnea, fatigue, chest pain, etc.) were recorded [30].

### 2.5. Statistical Analysis

SPSS statistical software, version 25, was used for statistical analysis. After using the Kolmogorov-Smirnov test to check the normality of the data distribution, differences between baseline and 3-month values were analyzed by paired two-sample Student's *t*-test. Differences between the groups were calculated by one-way analysis of variance (ANOVA) test. Data are expressed as mean ± SEM (standard error of mean). Significance level was defined as *p* < 0.05. The homogeneity of the groups was tested by Levene's *F*-test. The impact of parameters on 6MWT results was analyzed by the Pearson correlation analysis.

## 3. Results

### 3.1. Hemorheological Parameters

The apparent WBV was increased (*p* < 0.05) in heart failure patients, but Hct and PV did not show any difference in either resveratrol or placebo group compared to the control group at baseline. According to our results, resveratrol had no effect on Hct, PV, or WBV and no difference was observed between the two groups (RES and placebo) either at baseline or after the 3-month follow-up period (Table 2).

**Figure 2:**
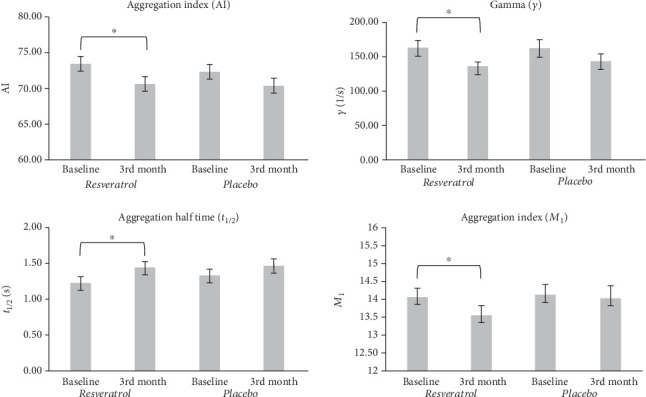
Effect of resveratrol on red blood cell aggregation. Values are expressed as mean ± SEM. Star (∗) = significant difference in 3^rd^ month values of the resveratrol group compared to the baseline values of the resveratrol group (*p* < 0.05). Baseline: measured values at randomization in the resveratrol or placebo group; 3^rd^ month: patients treated with resveratrol or placebo for 3 months; *M*_1_ value: aggregation index at 3 s^−1^ rotation speed of the Myrenne aggregometer; AI: aggregation index measured by LORCA aggregometer; *t*_1/2_ (s): aggregation half time; *γ* (1/s): threshold shear rate.

The AI and the threshold shear rate (*γ*) were significantly higher, and aggregation half time (*t*_1/2_) was significantly lower in both the resveratrol and placebo groups compared to the control group at baseline (*p* < 0.05). The *M*_1_ measured by Myrenne (*p* < 0.05), the LORCA AI (*p* < 0.05), and LORCA *γ* (*p* < 0.05) decreased significantly after RES treatment. Furthermore, *t*_1/2_ measured by LORCA also demonstrated a significant (*p* < 0.05) alteration after 3 months compared to baseline (Table 2, Figure 2).

The deformability of RBCs at any measured shear stresses did not show significant changes in either group after 3 months compared to baseline (Table 3), though an increasing tendency of EI at high shear stress could be seen in the resveratrol group.

### 3.2. Six-Minute Walk Test

The 6-minute walk distance improved significantly (275 ± 19.3 m vs. 298 ± 21.6 m, *p* < 0.05) after RES treatment compared to baseline but did not change in the placebo group (NS).

### 3.3. Relationship between 6-Minute Walk Distance and Hemorheological Variables

Hematocrit (*r* = 0.343, *p* < 0.05) and whole blood viscosity (*r* = 0.308, *p* < 0.05) had a moderate positive correlation with the 6MWT (correlation indices of the baseline pooled group). In the resveratrol group, significant correlation could be revealed between the 6-minute walk distance and the erythrocyte aggregation after 3 months: lower *M*_1_ aggregation index (*r* = −0.269, *p* < 0.05) and lower *γ* (-0.417, *p* < 0.05) were associated with longer walk distance. In addition, in this subgroup, significant positive correlation was found between 6MWT and EI at high shear stresses (EI30: *r* = 0.480, *p* < 0.005).

## 4. Discussion

The altered hemorheological factors in heart failure may play an important role in the complex impairment of microcirculation and the progression of heart failure. In previous studies, hemorheological parameters (WBV, plasma fibrinogen level, and RBC aggregation and deformability) were found significantly worse in heart failure patients than in healthy people [6]. Reduced peripheral blood flow, oxygen transport (hypoxemia), and increased oxidative stress in heart failure are described to cause RBC disorders [5, 6, 15]. Damage to red blood cells by ROS results in abnormalities in the function, morphology, and metabolism of erythrocyte including RBC aggregation and deformability [15, 31]. Cytoskeletal and membrane proteins and lipids are oxidized by ROS, which possibly increases the tendency of “damaged” erythrocytes to adhere with other erythrocytes thereby increasing RBC aggregation [32]. Our results demonstrated increased erythrocyte aggregation in heart failure patients compared to age-matched patients with moderate cardiovascular risk profile without heart failure (Table 2). Moreover, hypoxemia-induced rise in hematocrit (increased erythrocyte production) and elevated blood viscosity were observed in heart failure [6, 26], while in advanced stages of heart failure hematocrit level often decreases due to increased plasma volume (hemodilution), iron deficiency, and/or bone marrow depression caused by excessive cytokine and ROS production [33, 34].

According to literature, RES may have a protective role against the development of cardiovascular diseases. Primarily, RES is thought to be responsible for the cardioprotective effect of red wine (French paradox). The bioactive polyphenol resveratrol possesses antioxidant properties, reduces oxidative stress in animal models, and may contribute to the preservation of cardiac structure and function in animals [17–19]. Animal studies showed that RES can stabilize erythrocytes by the reduction of erythrocyte osmotic fragility [35]. RES was also described to maintain vascular endothelial function and to dilate blood vessels [19]. A previous human study of our working group assessing endothelial dysfunction detected significant improvement in vasorelaxation in RES-treated patients [8]. Other authors reported that resveratrol can change the properties of plasma proteins and preserve the structure of fibrinogen from conformational alterations [36–38], which may influence RBC aggregation as well.

The effects of resveratrol on hemorheological parameters have already been evaluated in some animal and human trials; however, they have not been studied in heart failure [8, 24].

The main findings of our present trial after a 3-month follow-up period are as follows.

(1) RBC aggregation was decreased significantly in patients treated with resveratrol; (2) macrorheological parameters did not change significantly; (3) the 6-minute walk distance was increased significantly in resveratrol-treated patients; (4) relationship was detected between the 6-minute walk distance and some hemorheological variables (erythrocyte aggregation, erythrocyte deformability, hematocrit, and whole blood viscosity).

We found no significant change in *Hct*, *WBV*, and *PV* values after the 3-month RES treatment. Similar results were seen in a previous study of our workgroup [8].

On the other hand, we could demonstrate a significant decrease of *RBC aggregation* in our patients after RES treatment via several parameters. Based on our results—which confirm a previous study [6]—RBC aggregation is deteriorated significantly in patients with heart failure, while in this study RES treatment partially reversed these changes of RBC aggregation. Though some difference could be seen between the results of LORCA and Myrenne, these are probably the consequences of the different principles of operation and precision of the instruments [25].

The decrease in aggregation may be a consequence of the antioxidant properties of RES and the modifications of plasma proteins as well. According to one of the accepted theories, RBC aggregation is due to the bridging between adjacent cells by specific plasma proteins (e.g., fibrinogen, large molecular weight globulins) and influenced by the concentration and specific binding to the erythrocyte membrane of fibrinogen. It is known that polyphenols are bound to plasma proteins due to their poor water solubility. RES may change the properties of plasma proteins and RBC surface molecules, thus reducing the ability to form cross-links between cellular components and decreasing erythrocyte aggregation [23, 37, 38]. The reduction of RBC aggregation may have a positive effect on the flow properties of coronary microcirculation, which can be especially important in heart failure.

In this trial, we could not demonstrate the change of *RBC deformability* after RES treatment by any ektacytometry parameters.

*6MWT* is a routine diagnostic procedure to quantify the exercise capacity of heart failure patients [39]. In this double-blind, randomized placebo-controlled study, resveratrol-treated patients had longer walk distance after the 3-month follow-up period, which is concordant with the results of a previous study [40]. Furthermore, a positive correlation was found between the functional capacity and the favorable hemorheological alterations as well. The improving hemorheological parameters may partly contribute to the longer walk distance in patients treated with resveratrol.

## 5. Conclusion

In our in vivo human study, we confirmed the beneficial effect of RES on erythrocyte aggregation in heart failure. The decrease of RBC aggregation by RES may positively influence the microcirculation, tissue perfusion, and oxygen supply, which may contribute to the improvement of the coronary and peripheral blood flow and probably increase the exercise capacity of patients with heart failure with reduced ejection fraction.

### 5.1. Limitations of the Study

This study has potential limitations. Although the study was double-blind, randomized, (1) the number of patients enrolled was low and the (2) follow-up period was relatively short. The rheological changes observed were significant (3) but the degree of change was modest.

Several positive effects of resveratrol by different mechanisms are proven. (4) It is not known exactly whether the positive effect of resveratrol on cardiac function and exercise capacity is due to these rheological changes or other mechanisms are also responsible for it. Further studies are required on heart failure patients to properly understand the exact biochemical and cellular mechanism of resveratrol.

## Figures and Tables

**Table 1:** Baseline characteristics of the study population according to the treatment arms.

	Resveratrol (*n* = 30)	Placebo (*n* = 30)
Age (year)	65.8 ± 1.9	67.5 ± 2.1
Male	22 (73%)	21 (70%)
Ejection fraction (%)	30.06 ± 1.04	31.70 ± 1.27
NT-proBNP (pg/ml)	2998 ± 507	3139 ± 446
Serum creatinine (*μ*mol/l)	99.77 ± 4.42	104.47 ± 4.82
Sys. BP (mmHg)	132.47 ± 3.4	128.77 ± 3.8
Dias. BP (mmHg)	79.1 ± 2.33	80.13 ± 2.66
Heart rate (beat/min)	72.2 ± 2.75	76.93 ± 2.5
*Etiological factors*		
Ischemic heart disease	17 (56.7%)	17 (56.7%)
Nonischemic (alcohol, chemotherapy, and myocarditis)	13 (43.3%)	13 (43.3%)
*Risk factors, comorbidities*		
Hypertension	22 (73%)	23 (76%)
Diabetes	13 (43%)	14 (46%)
Smoking	11 (36%)	8 (27%)
Pulmonary diseases (asthma, COPD)	7 (23%)	8 (27%)
BMI (kg/m^2^)	29.3 ± 0.9	30.4 ± 1.3
Target heart rate (>70/min)	23 (76.7%)	20 (66.7%)
Atrial fibrillation	7 (23%)	10 (33.3%)
*Concomitant treatment*		
ACE inhibitor/ARB	28 (93%)	29 (97%)
Beta-blocker	29 (97%)	28 (93%)
MRA	23 (76.7%)	21 (70%)
Ivabradine	6 (20%)	6 (20%)
*Diuretics*		
Loop diuretics (furosemide, etacrynic acid)	27 (90%)	28 (93%)
Thiazide or thiazide-like diuretics (hypothiazide, indapamide, etc.)	8 (27%)	9 (30%)
*Device therapy*		
CRT-P/D	9 (30%)	7 (23.2%)
ICD	4 (13%)	3 (10%)

Baseline characteristics of the study population according to the treatment arms. Values are expressed as mean ± SEM. There were no significant differences in characteristics between RES- (resveratrol-) and placebo-treated groups at baseline. ACEI: angiotensin-converting enzyme inhibitors; ARB: angiotensin receptor blocker; BMI: body mass index; COPD: chronic obstructive pulmonary disease; CRT-P/D: cardiac resynchronization therapy-pacemaker/defibrillator; Dias. BP: diastolic blood pressure; ICD: implantable cardioverter-defibrillator; MRA: mineralocorticoid receptor antagonist; NT-proBNP: N-terminal prohormone of brain natriuretic peptide; Sys. BP: systolic blood pressure.

**Table 2:** Effect of resveratrol on hemorheological parameters.

	Baseline			3^rd^ month	
	Control *N* = 20	Resveratrol *N* = 30	Placebo *N* = 30	Resveratrol *N* = 30	Placebo *N* = 30
Hematocrit (%)	43.35 ± 0.63	45.03 ± 0.98	44.93 ± 0.98	44.76 ± 0.93	44.10 ± 1.02
WBV (mPas)	4.04 ± 0.07	4.59 ± 0.13^#^	4.55 ± 0.14^#^	4.45 ± 0.13	4.42 ± 0.13
PV (mPas)	1.28 ± 0.02	1.34 ± 0.02	1.31 ± 0.02	1.32 ± 0.02	1.32 ± 0.02
*RBC aggregation (Myrenne)*					
*M*	5.91 ± 0.25	6.02 ± 0.36	6.38 ± 0.28	5.90 ± 0.31	6.36 ± 0.28
*M*_1_	13.77 ± 0.78	13.96 ± 0.42	14.14 ± .0.47	13.46 ± 0.40∗	13.84 ± 0.51
*RBC aggregation (LORCA)*					
AI	69.37 ± 1.1	73.36 ± 1.02^#^	72.08 ± 1.04^#^	70.55 ± 0.99∗	70.15 ± 1.14
*t*_1/2_ (s)	1.57 ± 0.11	1.23 ± 0.08^#^	1.34 ± 0.09^#^	1.45 ± 0.08∗	1.51 ± 0.11
*γ* (1/s)	112.75 ± 4.43	161.92 ± 11.02^#^	160.39 ± 12.54^#^	133.70 ± 7.25∗	138.60 ± 9.12

Values are expressed as mean ± SEM. ^#^Significant difference in the resveratrol or placebo groups compared to the control group at baseline; ∗significant difference in 3^rd^ month values of the resveratrol group compared to the baseline values of the resveratrol group (*p* < 0.05). Baseline: measured values at randomization in the resveratrol group or in the placebo group; 3^rd^ month: patients treated with resveratrol or placebo for 3 months; AI: aggregation index; *t*_1/2_ (s): aggregation half time; *M* and *M*_1_ values: aggregation indices at different rotation speeds of the aggregometer; PV: plasma viscosity; RBC: red blood cell; WBV: whole blood viscosity; *γ* (1/s): threshold shear rate.

**Table 3:** Effect of resveratrol on red blood cell deformability.

	Baseline		3rd month	
	Resveratrol (EI)	Placebo (EI)	Resveratrol (EI)	Placebo (EI)
Shear stress				
30 (Pa)	0.619 ± 0.003	0.623 ± 0.001	0.621 ± 0.002	0.623 ± 0.001
3 (Pa)	0.415 ± 0.004	0.420 ± 0.003	0.414 ± 0.004	0.422 ± 0.003
0.3 (Pa)	0.008 ± 0.009	0.009 ± 0.006	0.005 ± 0.007	0.009 ± 0.006

Values are expressed as mean ± SEM. There was no difference in the EI values at any shear stresses between the placebo, resveratrol baseline, and 3^rd^ month groups. Baseline: measured values at randomization in the resveratrol or placebo group; 3^rd^ month: patients treated with resveratrol or placebo for 3 months. EI: elongation index; Pa: pascal.

## Data Availability

The individual patient data used to support the findings of this study are available from the corresponding author upon request.
